# Graph Convolution Neural Network and Deep Q-Network Optimization-Based Intrusion Detection with Explainability Analysis

**DOI:** 10.3390/s26051421

**Published:** 2026-02-24

**Authors:** Kelvin Mwiga, Mussa Dida, Leandros Maglaras, Ahmad Mohsin, Helge Janicke, Iqbal H. Sarker

**Affiliations:** 1 School of Computational and Communication Sciences and Engineering, The Nelson Mandela African Institution of Science and Technology, Arusha 23311, Tanzaniamussa.ally@nm-aist.ac.tz (M.D.); 2 Department of Land Management and Valuation, Ardhi University, Dar Es Salaam 16103, Tanzania; 3 School of Computer Science and Informatics, De Montfort University, Leicester LE1 9BH, UK; 4 Centre for Securing Digital Futures, Edith Cowan University, Perth, WA 6027, Australia; a.mohsin@ecu.edu.au (A.M.); h.janicke@ecu.edu.au (H.J.); 5 Institute of Computer Science and Digital Innovation, UCSI University, Kuala Lumpur 56000, Malaysia

**Keywords:** GCN, Deep Q Network, attention mechanism, intrusion detection, explainable AI

## Abstract

As networks expand in size and complexity, coupled with an exponential increase in intrusions on network and IoT systems, this leads to traditional models failing to capture increasingly intricate correlations among network components accurately. Graph Convolution Networks (GCNs) have recently acquired prominence for their capacity to represent nodes, edges, or entire graphs by aggregating information from adjacent nodes. However, the correlations between nodes and their neighbours, as well as related edges, differ. Assigning higher weights to nodes and edges with high similarity improves model accuracy and expressiveness. In this paper, we propose the GCN-DQN model, which integrates GCN with a multi-head attention mechanism and DQN (Deep Q Network) to adaptively adjust attention weights optimizing its performance in intrusion detection tasks. After extensive experiments using the UNSW NB15 and CIC-IDS2017 dataset, the proposed GCN-DQN outperformed the baseline model in classification accuracy. We also applied LIME and SHAP techniques to provide explainability to our proposed intrusion detection model.

## 1. Introduction

Networked devices and their associated technologies have revolutionized how we live, work and manage our businesses. As the population increases tremendously, the need arises for more networked devices among people to facilitate communication. Increased network devices and their associated connections among computational devices have become a significant challenge in cyber security. As networks expand, the risk of cyberattacks escalates due to the increasing difficulty of monitoring them. In the real world, network devices and computer systems are susceptible to several cyberattacks in the highly digital world [1]. Network intrusion in IoT and other computer systems can have serious repercussions, causing financial losses, confidential data theft, or even significant infrastructure disruption. Network intrusion detection systems (NIDSs) are commonly viewed as a defensive mechanism to counter and alleviate intrusions within network traffic [2]. The NIDSs developed to capture malicious network traffic have repeatedly demonstrated their efficacy in detecting and protecting against cyberattacks. There are two types of known NIDS: anomaly detection and Signature-based detection. Signature-based Network Intrusion Detection Systems (NIDSs) analyse and compare incoming traffic flows against predefined attack signatures to detect known network threats. Signature-oriented Intrusion Detection Systems (IDSs) can effectively recognize attacks using previously established patterns. However, they fail miserably when identifying new attacks. Anomaly-based Network Intrusion Detection Systems (NIDSs) identify potential intrusions or threats by monitoring and analysing varied network patterns and activities, focusing on newly discovered or previously unknown network behaviours.

Machine learning (ML)-based intrusion detection systems (IDSs) are particularly noteworthy among IDSs, characterized by their capability to learn the data patterns and use learned patterns to detect any data pattern that deviates from vast volumes of data [3]. Although these models are remarkably effective at learning new patterns, one prevalent limitation in these ML-based IDS models is that they treat individual data samples without considering the interdependencies within a given network. Deep learning (DL), a crucial topic of ML, offers a promising solution to tackle this challenge with its superior feature learning capabilities.The prevailing advantage of a deep learning algorithm lies in its capability to automatically learn appropriate data patterns directly from massive datasets to generate models that correctly depict particular objects [4].

Numerous researchers have recently focused on DL approaches for handling graph-structured data [5]. A graph is a well-structured data representation capable of illustrating and extracting complex data patterns. Graphs depict entities (nodes) and their interactions (edges) in a structured, interconnected manner that incorporates a system’s local and global behaviours to provide a complicated system that will be harder for adversaries to evade. Indeed, network intrusions involve suspicious and benign data transfer streams between network hosts or within host system processes. Graph Representation Learning, employing models such as GNNs, enhances cybersecurity systems’ comprehension of relationship behaviour, resulting in more effective detection of threats, such as malware detection [6], threat intelligence [7], and vulnerability detection [8]. The three most popular GNN models include Graph Recursive Neural Networks (GRNNs) [9], Graph Attention Networks (GATs) [10], and Graph Convolutional Networks (GCNs) [11].

Drawing inspiration from recent studies in Graph Neural Network (GNN)-based approaches for extracting mutual feature correlations [11], this work proposes a novel hybrid anomaly IDS architecture that leverages graph-based convolution models (GCNs) and attention-based techniques. The GCN component captures complex, heterogeneous feature interactions, while the integrated attention mechanism improves the model’s capability to learn local and global dependencies within graph-structured data. Moreover, we used a reinforcement learning (RL) component in the model architecture to provide a feedback mechanism to enhance model performance by providing real-time feedback to the attention mechanism based on model predictions. This innovative architecture enhances the ability of anomaly IDS to detect complex, multi-dimensional patterns and relationships within network traffic data, resulting in more accurate and resilient intrusion detection. The key contributions of this study are outlined below:

- We introduce a hybrid architecture that combines GCN, attention mechanisms, and DQN to create optimal adaptive performance through reinforcement learning, enhancing real-time threat detection accuracy. DQN adaptively optimizes attention weights to optimize GCN attention model training.
- A unified approach framework is developed to enhance the performance of the network intrusion detection model through adaptive prioritization of features.
- The Explainable AI (XAI) concept is implemented to identify the most influential features and assess their impact on model detection performance using LIME and SHAP techniques. XAI offers transparency into how the GCN-based IDS makes decisions by revealing the role of each feature and its contribution to the final prediction.

The next sections of this research are organized as follows: In ‘Related works’, we discussed a comprehensive view of the concepts and techniques used in our research, including GCN, attention mechanism, reinforcement learning, and XAI; ‘The proposed model’ establishes the research methods of our proposed framework and its layered architecture in enhancing anomaly detection. ‘Experimental test results and discussion’ shows the evaluation and comprehensive analysis of the model’s experimental results, and finally, in ‘Conclusions’, we conclude and outline future work.

## 2. Related Works

The existence of malicious traffic in network traffic and the methods to identify anomalous network traffic from massive data flows have attracted many scholars. The advancement of artificial intelligence has led to machine learning, deep learning, and graph neural networks emerging as essential components for identifying malicious network traffic.

### 2.1. Machine Learning-Based Intrusion Detection Methods

Intrusion detection systems that utilize machine learning are capable of distinguishing anomalous traffic from normal network activity by analysing network traffic characteristics through diverse machine learning models. Remarkable performance in detecting anomalous network traffic is shown by machine learning models such as Random Forest (RF), Support Vector Machine (SVM), k-Nearest Neighbour (k-NN), and Gradient Boosting.

Ref. [12] proposed a network intrusion detection method integrating IGRF-RFE for feature selection. Experimental results based on UNSW NB15 reveal that the features were reduced from 42 to 23, while performance accuracy using the MPL algorithm increased from 82.25% to 84.24%. Ref. [13] proposed a malicious traffic detection approach that uses Random Forest, Decision Tree, Logistic Regression, K-Nearest Neighbors, and Artificial Neural Networks. On the UNSW-NB15 dataset, the model achieved 89.29% accuracy with the Random Forest algorithm. After applying the Synthetic Minority Over-sampling Technique (SMOTE) and selecting 24 selected features through Principal Component Analysis (PCA), the model’s accuracy improved to 95.1%.

The authors of [14] introduced IDS in 2023 based on a Gaussian mixture model and a one-class SVM. In the study, the one-class support vector machine (OCSVM) and the Gaussian mixture model (GMM) are trained on features generated after an autoencoder (AE) extracts representative features from normal data. Their performance on the IDS2018 dataset achieved an accuracy of 95.10%, but with a relatively high false positive rate (FPR) of 5.772%. Prasad et al. [15] proposed feature-focused multi-level correlation using the UNSW NB15 dataset. The proposed intrusion detection system used Pearson correlation and feature-to-label correlations in the two-level feature selection. The results show that 15 features were selected. The decision tree model in the multi-classification experiment obtained a classification accuracy of 95.2%.

Similarly, Ref. [16] examined techniques such as C4.5, Naive Bayes, Multi-Layer Perceptron (MLP), NB-Tree, and Decision Tree for detecting attacks, including DoS and Distributed Denial of Service (DDoS). The Decision Tree combined with Recursive Feature Elimination (RFE) achieved an accuracy of 99%, while the XGBoost algorithm, optimized using a parallel computing environment, attained a detection rate of 99.60%. Despite these encouraging results, the authors noted that traditional machine learning approaches have inherent limitations when compared to deep learning methods, which are better suited for modelling complex data patterns.

### 2.2. Deep Learning-Based Intrusion Detection Methods

Deep learning techniques show remarkably excellent capabilities in handling vast amounts of data in comparison to traditional ML methods. Since deep learning has become popular for identifying attack network traffic, Sun et al. [17] proposed an intrusion detection system called DL-IDS, a hybrid network that combines CNN and LSTM to extract spatial and temporal features of network data flow; however, its ability to identify certain attack types needs improvement. Zheng et al. [18] presented a Conv-LSTM framework for predicting traffic flow.

Despite these advances, deep learning methodologies predominantly focus on spatial and temporal features, often overlooking complex network relationships and lacking model transparency. Furthermore, the opaque and inherent complexity of deep learning models presents significant challenges for explainability and trust in cybersecurity operations. Addressing these challenges, Keshk et al. [19] proposed an explainable deep learning-based intrusion detection framework designed specifically for IoT networks, combining LSTM models with the SPIP explainability framework to improve detection performance and provide both local and global interpretability. Additionally, work in the study by Mohsin et al. [20] emphasizes the significance of explainable deep learning models to foster trust and transparency in AI-driven cybersecurity operations for threat and anomaly detection tasks.

### 2.3. Malicious Detection Methods Based on Graph Neural Networks

Graph Neural Networks (GNNs) have emerged as an increasingly prominent approach for network security research in recent years since they have an exceptional ability to analyse data in graph structure. Kipf et al. [21] introduced a scalable semi-supervised learning method based on GNNs that directly operated on the GNN structure utilizing an effective variation of Convolutional Neural Networks (CNNs) to enhance detection efficiency. A GNN-based anomaly detection algorithm was proposed byGuo et al. [22]. This algorithm trains the representation vector for anomaly identification by incorporating network structure, attributes, and dynamic change information into the model. Nevertheless, this technique needs to be improved in terms of operational efficiency. The E-minBatch model, developed from GraphSAGE, was presented in [23]. In this model, the source node is defined using the application layer’s source port and source IP address, while the target node is constructed from the target port and target IP address. The remaining traffic data is utilized as edge information to form the graph structure. Nevertheless, this method was restricted to the detection of industrial Internet traffic. To detect malware, Niu et al. [24] suggested an adaptive online analysis-based method that analyses encrypted, precisely drifting, and imbalanced network traffic. Diao et al. [25] suggested Graph Convolutional Networks (GCNs) that combined Conditional Random Fields and attention. They successfully predicted traffic flow using GCN’s node feature and structure information recognition capabilities.

## 3. The Proposed Model

**Figure 1:**
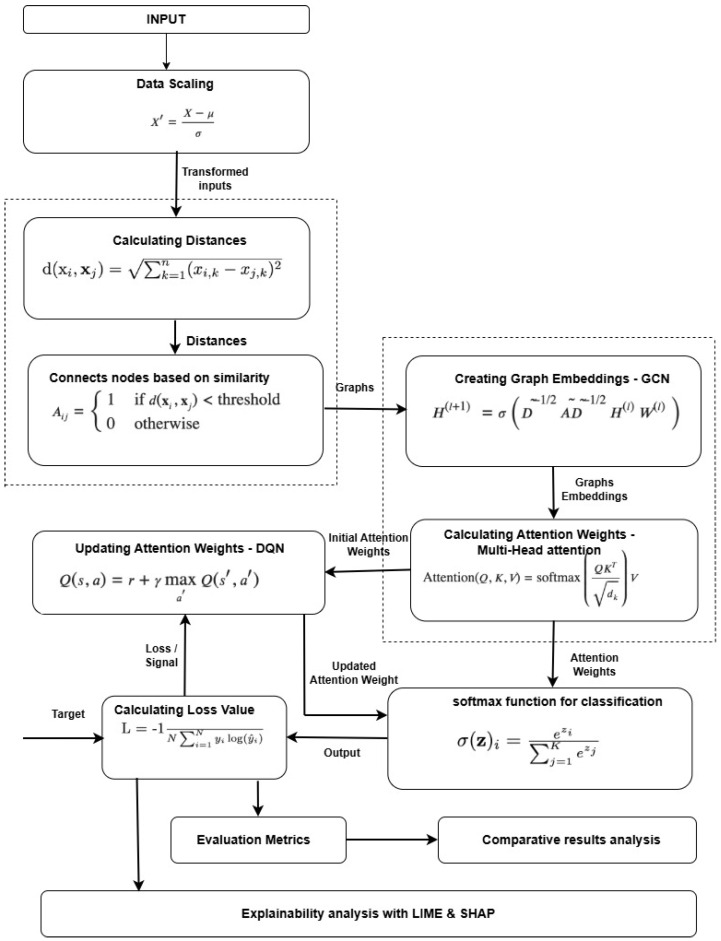
GCN-DQN methodology: An overview model to optimize GCN-based malicious detection with explainability.

To optimize GCN-based malicious detection, we propose a GCN-DQN technique that combines graph convolutional neural network (GCN), multi-head self-attention (MSA), and the Deep Q-Network mechanism to enhance GCN-based attack detection. Figure 1 illustrates the entire process of the proposed GCN-DQN approach.

### 3.1. Data Preprocessing and Feature Engineering

First, we focus on the basic data preprocessing processes such as data cleaning, hot encoding, resampling, and normalization on original datasets. Data preparation is essential to make the data understandable and learnable by the deep learning model before it is used. Datasets such as UNSW NB15 contain instances of missing and redundant data. When a class is insufficiently represented within a dataset, it becomes imbalanced. The intrusion detection system’s efficacy is diminished due to the difficulty of detecting the minority class. Since the UNSW NB15 and CIC-IDS2017 datasets are highly imbalanced, we employed SMOTEENN [26] only to the training dataset after splitting to address the class imbalance. It combines SMOTE and ENN methods. After using SMOTEENN, the final class distribution is benign (99,883), DoS (99,960) and Portscan (99,915) for the CIC-IDS2017 dataset. Likewise, the final class distribution for UNSW NB15 is benign (107,604) and attack (101,746).

Furthermore, the UNSW NB15 and CIC-IDS2017 datasets contain nominal features that are incompatible with the algorithms utilized in this study for model training. These categorical features must, therefore, be transformed into numerical features. Our study employed a label encoder to transform categorical data instances to numerical ones since GCN, Deep Q Network, and attention mechanism require numerical input and output variables. Upon examining each feature’s mean and standard deviation, significant variations exist among the numerical features that could produce biased results when using machine learning techniques. Our study used a standard scaler to standardize the dataset’s features within normalized ranges [0, 1]. Log1p was used to reduce skewness in the UNSW NB15 dataset.

Feature correlation was used to choose highly correlated features. This study used the feature correlation technique to measure the relationship between features and pick only those with moderately larger positive or negative values, i.e., closer to 1 or −1. The UNSW NB15 dataset’s correlation matrix shows that features such as sbytes and sloss are mostly correlated, and dbytes correlate strongly with dloss. Sbytes and dloss were dropped since the two features are highly correlated. Similarly, there was no correlation between features in the CIC-IDS2017 dataset.

**Features Selection: Mutual Information Strategy**

We used a mutual information method in our feature selection process [27]. We computed mutual information scores for both the CIC-IDS2017 and UNSW NB15 datasets. SelectKBest, part of the sklearn library, was used to select 38 and 65 features for the UNSW NB15 and CIC-IDS2017 datasets, respectively.

### 3.2. Graph Construction Using Euclidean Distance-Based Adjacency Matrix

First, the distance between network traffic data instances was calculated using the Euclidean distance [28] shown in Equation (1).

The Euclidean distance between two vectors $\left(x_{i}\right)$ and $\left(x_{j}\right)$ is given by:

(1) $$d\left(x_{i},x_{j}\right)=\sqrt{\sum_{k=1}^{n}{\left(x_{i,k}-x_{j,k}\right)}^{2}}$$

This study uses the adjacency matrix (A) to show the presence of edges connecting pairs of nodes using the calculated Euclidean distance between data point instances. If $A_{ij}=1$, there is an edge between nodes I and J. If not, $A_{ij}=0$.

$$A_{ij}=\left\{\begin{array}{cc} 1 & \mathrm{if}\;d(x_{i},x_{j})<\mathrm{threshold}\\ 0 & \mathrm{otherwise} \end{array}\right.$$

### 3.3. Graph Embeddings Creation

By using convolution operations on graphs, GCNs may find hierarchical patterns in network data. This capability is crucial for detecting complex traffic patterns and potential cybersecurity threats. A multi-layered GCN can directly process data structured as a graph and leverage the attributes of neighbouring nodes to guide the learning of each node’s embedding vector, thereby enabling the extraction of feature representations for previously unseen nodes. Multi-layered GCN can directly handle data in a graph structure and use the attributes of nearby nodes to guide each node’s embedding vector learning, thereby acquiring the feature representations of new nodes and detecting anomalous behaviour, enhancing the comprehensive capture of relevant information in the detection. The GCN layer captures local graph structures by aggregating features from a node’s immediate neighbours [29]. The input and output node representations for the lth graph convolution layer are represented by the matrices $H^{\left(l-1\right)}$ and $H^{\left(l\right)}$, respectively [30]. The first node is shown by the original input feature $\left(H^{\left(0\right)}\right)$ = X, which is usually configured to show the first graph convolution layer’s input. The graph convolution operation of the input feature matrix $\left(H^{\left(0\right)}\right)$ provides the output of the first layer of the GCN $\left(H^{\left(1\right)}\right)$, as illustrated in Equation (2).

(2) $$H^{\left(1\right)}=\mathrm{ReLU}\left(MXW^{\left(0\right)}\right)$$

In Equation (2), the normalized adjacency matrix M [4], which includes nodes with self-loops, is incorporated for each applied node and considered during the normalization of the adjacency matrix, as illustrated in Equation (3).

(3) $$M={\tilde{D}}^{-\frac{1}{2}}\tilde{A}{\tilde{D}}^{-\frac{1}{2}}$$

In Equation (3), $\tilde{A}=A+I$, $\tilde{D}$ is the degree of the matrix related to $\tilde{A}$ and $W^{\left(0\right)}$ is the specific learnable weight matrix at layer l. The ReLU function in Equation (4) is a non-linear activation function that makes the model non-linear [4].

(4) $$H^{\left(l+1\right)}=ReLU\left(MH^{\left(l\right)}W^{\left(l\right)}\right)$$

### 3.4. Attention Weights Calculation

Across a range of tasks, attention mechanisms are essential for enhancing model efficiencies [31]. Self-attention facilitates the automatic selection of relevant information based on input data, effectively capturing long-range dynamic dependencies tasks. We carry out multi-head attention using the scaled dot-product suggested in Vaswani [31]. The result of single-head attention is computed as

(5) $$\mathrm{Attention}\left(Q,K,V\right)=\mathrm{softmax}\left(\frac{QK^{T}}{\sqrt{d_{k}}}\right)V$$

The scaled dot-product technique helps the model better grasp the entire network structure during learning by using its strength in integrating global information to allocate attention weights dynamically among nodes. In practice the scaled dot-product technique maps the query, key–value pairs, and output vectors. First, the feature input vector from input layer $H_{i}$ is multiplied by weight matrices of $W_{q}$, $W_{k}$ and $W_{v}$, giving rise to query (Q), key (K), and value (V). Mathematically, it can be represented as shown in Equation (6).

(6) $$\begin{array}{cc} Q & =H_{i}\times W_{q},\\ K & =H_{i}\times W_{k},\\ V & =H_{i}\times W_{v}. \end{array}$$

The dot products of query and keys, multiplied by $\frac{1}{\sqrt{d_{k}}}$, are integrated with the softmax function to obtain the weight of the value. Finally, the value v and the calculated weight of the value are multiplied to obtain the attention score $\left(\frac{QK^{T}}{\sqrt{d_{k}}}\right)$. The $d_{k}$ represents the dimensionality of the key/query vectors, whereas T represents transpose.

### 3.5. Model Classification and Loss Value Calculation

The classification layer of our model utilizes a linear transformation, taking attention weights from the self-attention layer as input features, and outputs the predicted influence values of the nodes. After classification, the model computes the loss between the predicted values and the corresponding targets. The loss function, cross-entropy loss for the classification, is presented in Equation (7).

(7) $$L_{\mathrm{CE}}=-\sum_{i}y_{i}\log\left(p_{i}\right)$$

where $\left(y_{i}\right)$ is the true label and $\left(p_{i}\right)$ is the predicted probability for class (*i*).

### 3.6. Optimizing Attention Weights via Deep Q-Learning

The attention mechanism plays a critical role in determining which node-to-node relationships within the graph structure are emphasized during message passing. However, fixed or purely backpropagation-learned attention weights may not always reflect the most discriminative structural patterns in dynamic intrusion behaviour. To address this limitation, we integrate a Deep Q Network (DQN) [32] that adaptively adjusts the attention weights based on the model’s ongoing classification performance. A reinforcement learning (RL) [33] layer is employed with the graph attention mechanism, allowing the model to explore alternative attention configurations and converge on those that minimize classification loss.

The optimization process is triggered whenever the cross-entropy loss exceeds a predefined threshold. Instead of relying directly on backpropagation to adjust the attention parameters, the system uses DQN, treating the attention configuration as part of a sequential decision-making problem to improve predictive performance.

#### 3.6.1. RL State Representation

At time step (t), the DQN receives a state vector that summarizes both the current attention configuration and the classification performance of the GCN. The state is expressed as:

(8) $$s_{t}=\left[A_{t};\;\mu \left(H_{t}\right);\;\sigma \left(H_{t}\right);\;L_{t}\right]$$

where:

- $A_{t}$—the current attention weight matrix. This matrix determines how strongly each neighbouring node contributes to the node update function.
- $\mu (H_{t})$—the mean of the node embeddings output by the GCN. It reflects the centre of the latent graph representation.
- $\sigma (H_{t})$—the variance of the node embeddings. It indicates how diverse or homogeneous the node representations are within the graph.
- $L_{t}$—the current cross-entropy classification loss.

Together, these components allow the DQN to assess not only how the model is focusing (via attention) but how well it is performing (via loss and embedding statistics).

#### 3.6.2. Action Space for Attention Adjustment

DQN operates on discrete actions. We therefore define a discrete action set:

$$A=\{a_{1},\;a_{2},\;a_{3},\;a_{4}\}$$

Each action modifies the attention weights by a small step size α = 0.05:

- $a_{1}:\;A_{t+1}=A_{t}+\alpha$ (increase attention weights);
- $a_{2}:A_{t+1}=A_{t}-\alpha$ (decrease attention weights);
- $a_{3}:A_{t+1}=A_{0}$ (reset to baseline);
- $a_{4}:A_{t+1}=A_{t}$ (no change).

This discrete set enables systematic exploration without destabilizing the GCN.

#### 3.6.3. The Reward Function

The reward measures how much the classification loss improves after applying an action. It is defined as:

(9) $$r_{t}=L_{t-1}-L_{t}$$

This reward formulation implies:

- Positive reward if the new loss is smaller → beneficial attention adjustment.
- Negative reward if the loss increases → harmful adjustment.
- Zero reward if the loss remains unchanged → neutral adjustment.

This direct use of loss reduction as the reward signal tightly couples RL decisions with classification performance.

#### 3.6.4. State Transition Dynamics

After executing action at, the attention matrix becomes:

$$A_{t+1}=A_{t}+a_{t}\cdot \alpha$$

With the updated attention weights, the GCN recalculates the node embeddings and loss. The next state is therefore:

(10) $$s_{t+1}=f\left(A_{t+1},H_{t+1},L_{t+1}\right)$$

where f(·) captures:

1. The application of updated attention in the attention-weighted GCN layer.
2. The forward pass through the classification head.
3. The computation of new loss $L_{t+1}$.

#### 3.6.5. GCN Forward Pass Under Attention Control

The RL-modified attention weights affect message passing. For each pair of neighbouring nodes (i) and (j), the attention score is computed as:

(11) $$e_{ij}=a\left(Wh_{i},Wh_{j}\right)$$

where (a(·)) is the attention scoring network and (W) is a learnable weight matrix.

These scores are normalized using softmax:

(12) $$\alpha_{ij}=\frac{\exp(e_{ij})}{\sum_{k\in N(i)}\exp\left(e_{ik}\right)}$$

Node i’s updated embedding is then computed as:

(13) $$h_{i}^{\prime }=\sum_{j\in N(i)}\alpha_{ij},Wh_{j}$$

Since $A_{t}$ scales the $\alpha_{ij}$ coefficients, the DQN directly influences which neighbours the GCN listens to.

#### 3.6.6. Deep Q-Learning Update

The Q-value update equation is based on the Bellman equation:

(14) $$Q\left(s_{t},a_{t}\right)=r_{t}+\gamma \max_{a^{\prime }}Q\left(s_{t+1},a^{\prime }\right)$$

To learn this function, the DQN minimizes the temporal-difference (TD) loss [32]:

(15) $$L\left(\theta \right)={\left(r_{t}+\gamma \max_{a^{\prime }}Q\left(s_{t+1},a^{\prime };\theta^{-}\right)-Q\left(s_{t},a_{t};\theta \right)\right)}^{2}$$

where:

θ—parameters of the online Q network;

$\theta^{-}$—parameters of the target network (updated periodically for stability);

γ—discount factor that controls the weight of future rewards.

This formulation enables stable convergence to an optimal attention-modification policy.

All parameters, including GCN weights, attention parameters, and DQN weights, are optimized jointly using the Adam optimizer.

## 4. Experimental Test Results and Discussion

This section presents a series of comprehensive experiments performed on multiple datasets to evaluate the effectiveness of the proposed approach.

### 4.1. Datasets

This study evaluates the proposed model using two up-to-date and widely recognized datasets, CICIDS2017 and UNSW-NB15, which contain a diverse range of labelled attack and benign network flows. Initially, raw data is collected from network traffic generated by devices such as routers and switches. This data is then processed through specific techniques to produce structured dataset flows. These datasets play a critical role in identifying malicious behaviours and attacker activities during cyberattacks, serving as the foundation for both training and evaluating deep learning models. Therefore, selecting high-quality datasets that align with the model’s objectives is essential for achieving reliable and meaningful results.

#### 4.1.1. UNSW NB15

The Australian Centre for Cyber Security (ACCS) Cyber Range Lab released the UNSW-NB15 dataset by Moustafa and Slay (2015) [34], including normal and attack network behaviours. The dataset includes 2,218,761 benign flows (87.35%) and 321,283 attack flows (12.65%). The flows are classified into 10 classes: benign, analysis, backdoor, DoS, exploits, fuzzers, generic, reconnaissance, shellcode, and worms. Data preprocessing is important due to a substantial imbalance between normal and attack flows. We used 209,350 flows from the processed dataset for the experiment, including 107,604 normal flows and 101,746 malicious flows. The dataset contains 49 features.

#### 4.1.2. CIC-IDS2017

The dataset reflects real-world scenarios and includes diverse traffic samples from modern attacks [35]. The CIC-IDS2017 dataset exhibits class imbalance, with minority attack categories such as Web Attack, Bot, and Infiltration accounting for only 0.88%, 0.79%, and 0.01% of the total data, respectively.

### 4.2. Metrics for Evaluation

To evaluate the efficacy of our GCN-DQN cybersecurity model, we compute the results based on precision ($P_{r}$), recall ($R_{c}$), F-score ($F_{1}$), ROC value, and overall accuracy ($A_{c}$), as detailed below [29]. These measures have been extensively employed in many publications (e.g., Guojia-Yan et al. [22], Chen et al. [29]) to evaluate model efficacy in both multiclass and binary classification.

(16) $$A_{c}=\frac{TP+TN}{TP+FP+TN+FN}\times 100\%$$

(17) $$P_{r}=\frac{TP}{TP+FP}\times 100\%$$

(18) $$R_{c}=\frac{TP}{TP+FN}\times 100\%$$

(19) $$F_{1}=2\times \frac{\mathrm{Precision}\times \mathrm{Recall}}{\mathrm{Precision}+\mathrm{Recall}}\times 100\%$$

Here, TP represents true positives, FP denotes false positives, TN signifies true negatives, and FN indicates false negatives.

### 4.3. Experimental Settings

We used the Pytorch framework version 2.8.0 to build our proposed model, and it was compiled with GPU support. Our study was conducted on a system equipped with an 8th Generation Intel(R) Core(^TM^) i7-8550U CPU running at 1.80 GHz (up to 1.99 GHz) and 16 GB of RAM. The study was performed on the Kaggle platform. The dataset was partitioned into training and testing subsets, with 70% allocated for training and the remaining 30% reserved for testing.

During the model training stage, various hyperparameters were selected and examined in order to find appropriate values that are ideal for the model. Adopting an expert tuning search strategy to fine-tune training parameters, we explored a wide range of optimal parameters. The parameters include learning rates of $1\times 10^{-3}$ and $1\times 10^{-3}$, weight_decay values of $1\times 10^{-2}$ and $1\times 10^{-2}$, a number of epochs of 250, early_stopping_patience values of 10 and 20 and the batch sizes of 32 and 16 for UNSW NB15 and CIC-IDS2017, respectively. In addition, other parameters include gamma (DQN) = 0.99, epsilon (DQN) = 1.0, epsilon_min (DQN) = 0.01 and adam optimizer. The rest of the proposed model’s hyperparameter settings are illustrated in Table 1.

### 4.4. Experimental Results

This subsection presents a comprehensive evaluation of the experimental results obtained from the proposed model for network traffic classification using the UNSW NB15 and CIC-IDS2017 datasets. A baseline model consists of GCN with a multi-head attention mechanism without a DQN component. The baseline performance of each model is compared against the performance of the same model enhanced with the integration of the Deep Q-Network (DQN) algorithm. The presence of DQN in our proposed GCN-DQN model helps update the model’s attention weights. First, binary classification experiments are implemented to demonstrate how to improve performance in distinguishing benign from malicious network flows. We present the results of multiclass classification experiments we conducted to evaluate the effectiveness of our GCN-DQN model in identifying various attack types.

#### 4.4.1. The Evaluation of Binary Classification Performance

We tested the proposed method applied to the UNSW-NB15 dataset with several experiments to see how well the proposed approach proved effective. Since the original UNSW NB15 dataset was so big, we chose a random sample of 209,350 instances. We divided the data examples into two categories: training and evaluation. A total of 70% of the dataset instances were allocated for training, while 30% were designated for testing. Table 2 demonstrates how well the proposed approach performed at binary classification with respect to accuracy, precision, recall, and $F_{1}$ score.

The experimental results in Table 2 demonstrate that the proposed GCN-DQN model was 97% accurate, while the baseline model achieved 92% accuracy. The proposed model outperforms the baseline model across multiple evaluation metrics, including recall, precision, and F1 score, as shown in Table 2.

**Figure 2:**
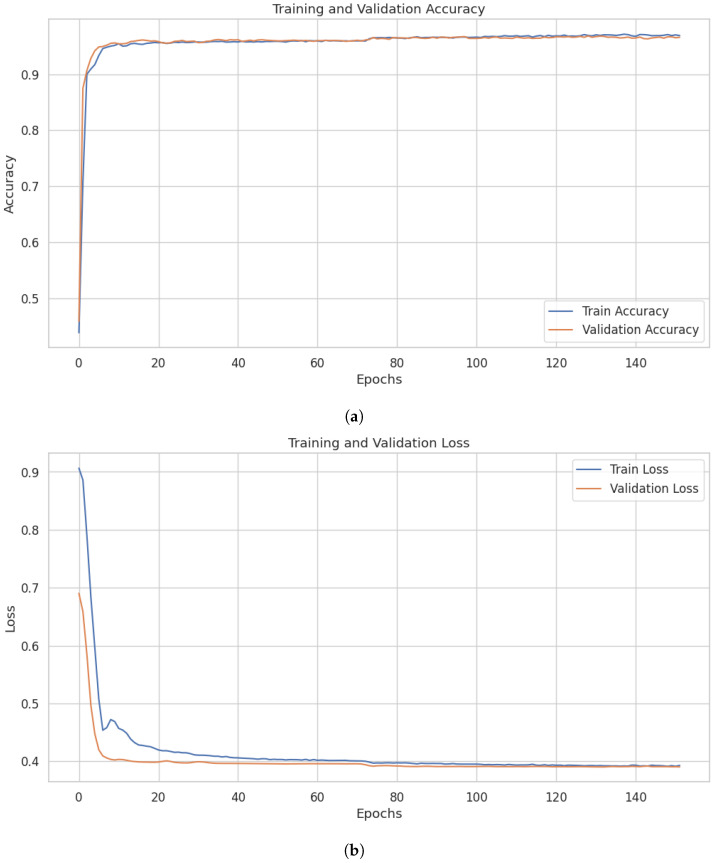
Accuracy and loss value curves with iteration step: (**a**) Model’s training and validation accuracy on UNSW NB15; (**b**) Training and validation losses of the model on the UNSW-NB15 dataset.

Figure 2a presents model training and validation/test accuracy. The figure shows the training and validation accuracy trends across epochs, providing insight into the model’s learning behaviour and generalization performance. It demonstrates that the initial training and validation/test accuracy was unstable. After 10–15 epochs, it achieved considerable stability in the model’s performance and generalization ability. The minimal gap between training and validation accuracies, especially from epoch 10 onward, indicates that the model exhibits strong generalization capabilities to unseen data.

Figure 2b illustrates the training and validation loss trends over 140 epochs. Figure 2b illustrates that the training and validation losses were initially unstable. It became relatively stable on 40 epochs. The model shows stable convergence, with losses decreasing and stabilizing at relatively low values (around 0.4).

#### 4.4.2. The Analysis of Multi-Classification’s Performance

The suggested technique distinguishes various network flows, as demonstrated in multiclass classification experiments. Table 3 presents the performance comparison between the proposed GCN-DQN model and the baseline mode. The proposed GCN-DQN model attained an accuracy of 99.02%, while the baseline model had an accuracy of 92.27%. The proposed model demonstrates superior precision, recall, and F1 scores compared to the baseline models. Table 3 shows the rest of the results.

**Figure 3:**
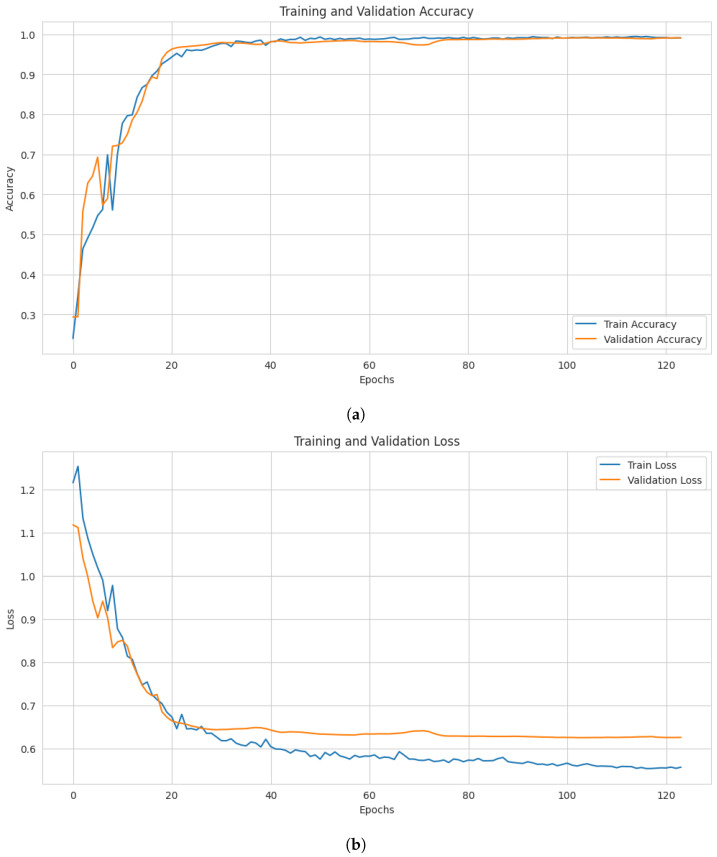
Accuracy and loss value curves with iteration step: (**a**) Model’s training and validation accuracies on CIC-IDS2017; (**b**) Model’s training and validation losses on CIC-IDS2017.

The relationship between the number of epochs in the proposed model and both the training and validation accuracies is illustrated in the accuracy plot shown in Figure 3a. The model achieved stable training and validation accuracies around 20 epochs. The model generalizes well since validation accuracy closely matches training accuracy. The model’s performance is relatively consistent across both the training and test datasets.

Furthermore, Figure 3b represents the model loss in training and test datasets against the epochs. The model demonstrates that the training and validation test’s losses were unstable at first, but they became much more stable around epoch 20. The model shows stable convergence with good generalization.

**Figure 4:**
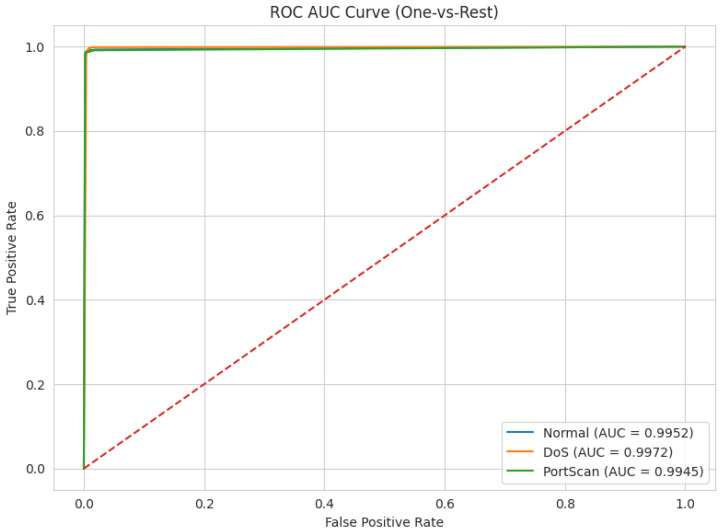
ROC-AUC curve for the CIC-IDS2017 dataset.

We analysed the receiver operating characteristic (ROC) curve to illustrate the relationship between the proposed model’s true positive rate (TPR) and false positive rate (FPR). The ROC-AUC metric is crucial for analysing the effectiveness of a deep learning models in detecting or classifying various attack scenarios, particularly for recognizing types of intrusions and attacks, as stated in this article. Figure 4 depicts the ROC-AUC, with True Positive Rate (TPR) plotted on the vertical axis (y-axis) and False Positive Rate (FPR) shown on the horizontal axis (x-axis). The diagonal dashed line represents the line of no discrimination, in which the True Positive Rate (TPR) equals the False Positive Rate (FPR). It depicts the performance of a random classifier with an AUC of 0.5. A higher AUC signifies enhanced model classification performance. Our proposed model obtained an excellent AUC of 0.9952 for normal flow and an AUC of 0.9972 for DoS, while Portscan attained an AUC of 0.9945, as seen in Figure 4.

### 4.5. Time and Memory Consumption

The performance evaluation of our GCN-DQN model involves a comprehensive analysis of inference time, training time, and memory usage to assess its suitability for real-world deployment. The proposed model is evaluated against these metrics using the UNSW NB15 and CIC-IDS2017 datasets to provide insight into its efficiency, responsiveness, and resource demands in practical intrusion detection scenarios. To calculate time and memory consumption, we used a memory-constrained web-hosted server with 4 GB RAM, 1 vCPU, 50 GB NVMe disk space, and 4 TB bandwidth. The results show that for CIC-IDS2017, the inference time for a batch of 128 samples is 0.017866, corresponding to a per-sample inference time of approximately 0.14 milliseconds, while UNSW NB15 achieves an inference time of 0.536309 for the same batch, corresponding to a per-sample inference time of 4.2 milliseconds. These low-inference time values indicate that our proposed GCN-DQN model can process network traffic efficiently, supporting timely detection and response to intrusions, and making it well-suited for near-real-time detection, especially for CIC-IDS2017, where inference is extremely fast.

In the analysis of the training time, the model required 44.98 s per training batch of 128 samples on the UNSW-NB15 dataset, which is substantially higher than the 0.24 s observed for CIC-IDS2017. This difference may be attributed to variations in dataset characteristics, such as feature distributions and graph structures, which may increase the computational overhead during training for UNSW-NB15. The GCN-DQN model is optimally designed for offline training with online inference, consistent with real-world intrusion detection practices. Memory consumption analysis further shows that the model required 3080 MB per batch for UNSW-NB15 and 4326 MB for CIC-IDS2017. The higher memory consumption observed for CIC-IDS2017 is likely attributable to increased feature dimensionality or greater graph complexity. Overall, the proposed model is computationally suitable for deployment on modern GPU-enabled systems and resource-constrained servers.

### 4.6. Comparison Analysis

We tested the GCN-DQN classifier on two datasets to achieve numerous classification results. The experimental analysis focuses on the proposed model’s binary and multi-classification results.

First, we compared the proposed model’s performance with reinforcement learning and without reinforcement learning components (baseline model) on the UNSW NB15 and CIC-ICD2017 datasets. The results from both the proposed GCN-DQN model and the baseline model were utilized. The findings from the proposed GCN-DQN model are contrasted with the baseline model in Table 2 and Table 3 for binary and multi-classification, respectively. As shown in Table 2 and Table 3, this comparison shows that the suggested model has reasonably improved performance with respect to accuracy, precision, recall, and F1 score. In the binary classification experiment, the proposed model achieved a 5% improvement in accuracy compared to the baseline model. The multi-classification experiment provided evidence that the proposed model attained an accuracy improvement of around 7% compared to the baseline model. Furthermore, the results are compared with state-of-the-art techniques commonly used in network intrusion detection, as shown in Table 4. Notably, our proposed model obtained better performance in both F1 score and accuracy. Our research also differs from previous studies by including explainability, which was not part of other studies.

### 4.7. XAI Analysis Based on SHAP-LIME

The study of XAI focuses on developing methods and algorithms that allow AI models to give human-comprehensible explanations for their decisions and predictions [41]. The concept of XAI is defined as a method to enhance human comprehension of AI decision-making processes [42]. Explainable AI aims to ease user understanding and interpretation of AI models [43,44]. Shapley Additive Explanations (SHAP) and Local Interpretable Model-Agnostic Explanations (LIME) were employed in the implementation to enhance the comprehensibility and transparency of the decision-making process in this study.

The SHAP summary plot is a visualization tool that illustrates the influence of various features on the output of a deep learning model. The vertical axis of the two-dimensional chart illustrates the feature names, while the horizontal axis represents the impact of SHAP values on the model’s output. The SHAP values along the horizontal axis reflect the degree to which each feature contributes to the model’s output.

**Figure 5:**
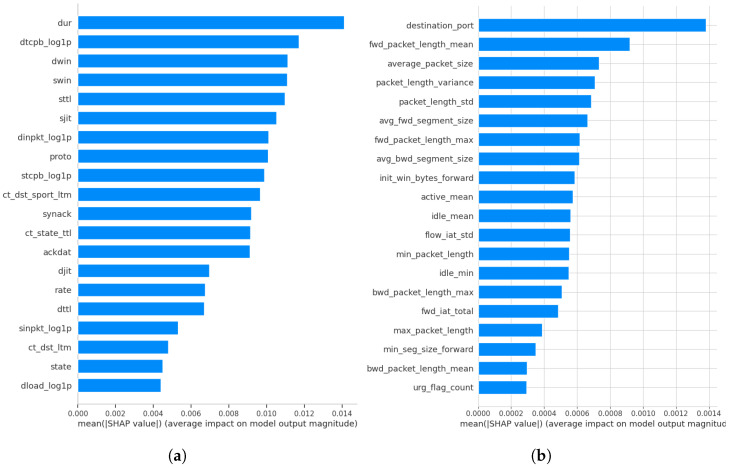
The summary in hierarchy of the features contributing to classification on (**a**) UNSW NB15 and (**b**) CIC-IDS2017.

Figure 5a,b depict the significant features with the highest scores obtained from the SHAP methodology applied to the UNSW-NB15 and CIC-IDS2017 datasets, respectively. The strength of each feature is determined by the magnitude of the SHAP value, with higher absolute SHAP values having a more significant influence on the model’s predictions. Insights from Figure 5 for binary and multi-classification highlight the importance of specific features with significant SHAP scores towards the model prediction. In Figure 5a, the network feature ‘dur’ has the highest SHAP score, signifying its paramount influence on network intrusion detection. This feature represents the duration of a network flow and plays a critical role in identifying anomalous connection behaviours, making it an essential attribute for detecting various attack types. The feature ‘dtcpb’ contributes highly after the feature ‘dur’ in Figure 5a. The ‘dtcpb’ feature captures the destination host’s initial TCP sequence number and assists in identifying abnormal TCP session initialization behaviour, making it useful for detecting protocol-based attacks. The ‘dload’ feature contributes least to our model’s classification performance, having the lowest observable SHAP value. It is noted that this feature examines network traffic patterns by monitoring the data transfer rate from the source to the destination, detecting unusual or potentially malicious activity.

Likewise, Figure 5b shows the summary of CIC-IDS2017 SHAP values, identifying each feature’s impact towards the model’s classification decision. The ‘destination_port’ feature achieved the highest SHAP value, which significantly impacts network intrusion detection. It allows the intrusion detection system to recognize the target service associated with each network flow, thereby enabling the model to differentiate between normal and malicious traffic by capturing port-specific usage and attack behaviours.

The ‘fwd_packet_length_mean’ feature shows moderate contribution to the model’s classification decision. ‘fwd_packet_length_mean’ represents the average size of packets sent in the forward direction of a network flow, allowing the IDS to detect abnormal sender-side traffic patterns linked to reconnaissance, brute-force, and denial-of-service attacks. The ‘urg_flag_count’ feature is observed to have the smallest SHAP value, meaning it has the least significant contribution to the model’s decision. The urg_flag_count feature enables the IDS to detect low-level TCP flag anomalies that are frequently associated with reconnaissance and attack-related traffic.

**Figure 6:**
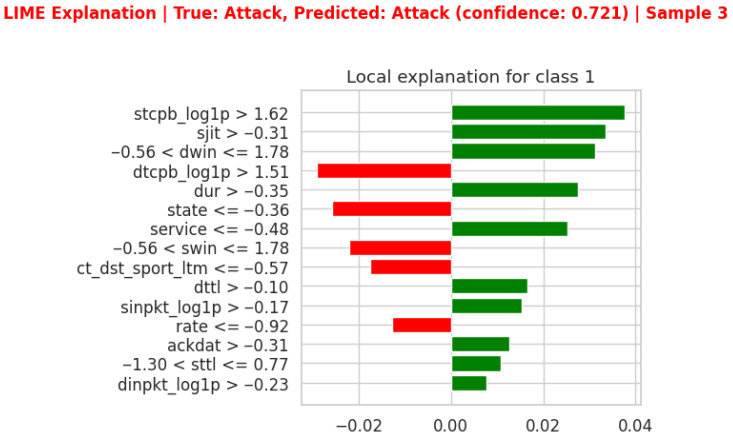
Feature importance scores using the LIME technique for binary classification.

LIME plots visualize the positive and negative contributions of each feature to the model’s decision-making, offering insights into why the proposed model produced specific predictions. The LIME diagram in Figure 6 illustrates the ten most important features that determine whether the network is normal or under attack at a particular data instance. A green horizontal bar appearing on the right of the plot indicates positive effects on the model’s decision-making. In contrast, a red bar extending to the left implies a negative influence. The lengths of these bars show how each feature contributes to the model’s decision-making. Figure 6 of the LIME explanation illustrates that features such as ‘stcpb > 1.62’, ‘sjit > −0.31’, ‘−0.56 < dwin <= 1.78’, ‘dur > −0.35’, ‘service <= −0.48’, ‘dttl > −0.10’, ‘sinpkt > −0.17’,‘ackdat > −0.31’, ‘−1.30 < sttl <= 0.77’ and ‘dinpkt > −0.23’ have values indicating a positive impact on the prediction. Conversely, features like ‘dtcpb > 0.51’, ‘state <= −0.36’,‘−0.56 < swin <= 1.78’, ‘ct_dst_sport_ltm <= −0.57’ and ‘rate <= −0.92’ have values suggesting a negative impact on the prediction.

SHAP local interpretation illustrates feature attributions, including Shapley values, as forces. SHAP local explanations focus on individual instances, generating explanations that indicate which feature values contribute positively and which contribute negatively to the model’s decision-making process. SHAP computes Shapley values to quantify the contribution of each feature to the model’s predictions [45]. In our analysis, we selected a specific instance and calculated its corresponding SHAP values.

**Figure 7:**
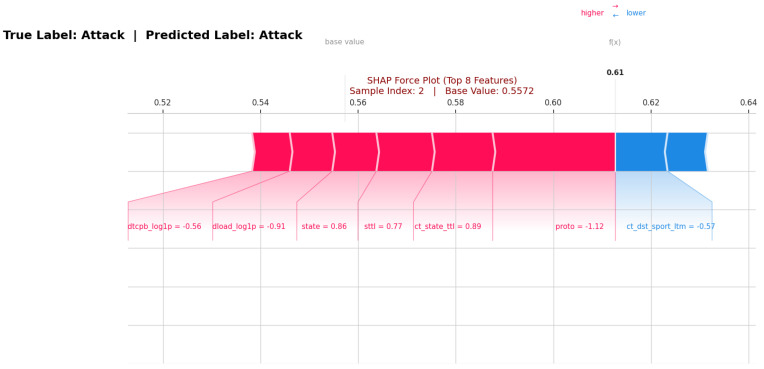
A local explanation of the attack class in UNSW-NB15.

Figure 7 displays the observation made with the UNSW-NB15 dataset, in which our deep learning (DL)-based IDS accurately predicted an attack as an attack. In this SHAP plot, we observe that the red features such as ‘proto = −1.12’, ‘ct_state_ttl = 0.89’, ‘state = 0.86’, ‘sttl = 0.77’, ‘dload = −0.91’ and ‘dtcpb = −0.56’ determine the likelihood for a data sample to be an attack. Conversely, blue features such as ‘ct_dst_sport_ltm = −0.57’ are forcing the classification lower, i.e., non-attack.

## 5. Discussion

This section reviews a series of experimental results regarding our GCN-DQN model, highlighting the proposed model’s contributions, strengths and interpretability.

The proposed GCN-DQN model with a Deep Q Network effectively adaptively optimizes attention weights to optimize GCN attention model training, boosting classification metrics across anomaly detection algorithms. Notably, our proposed model, evaluated on the publicly available UNSW-NB15 and CIC-IDS2017 datasets, did better than the baseline model with respect to accuracy, precision, F1 score, and recall. Using the UNSW NB15 dataset, the proposed model was 5% more accurate than the baseline model. Also, when we used the CIC-IDS2017 dataset, the proposed model was 7% more accurate than the baseline model.

Integrating GCN, attention mechanisms, and a Deep Q Network (DQN) provides a robust approach to create an optimal adaptive performance through reinforcement learning, enhancing anomaly detection. The experimental tests reveal that the suggested model worked well with the DQN component, getting 99.02% and 97% accuracy on the CIC-IDS2017 and UNSW NB15 datasets, as seen inTable 3 and Table 2 respectively. Our evaluation indicates that the GCN-DQN model surpasses other leading techniques in the realm of intrusion detection. In future work, we will apply the developed model in the SME domain [46].

Furthermore, the proposed model employs SHAP and LIME techniques to interpret the significance of features that played a critical role in detecting attacks, using the CIC-IDS2017 and UNSW-NB15 datasets. The application of SHAP and LIME techniques on our proposed model enhanced model explainability, as outlined in Section 4.7. These features can clarify the decision-making process of the proposed classifier and improve the comprehension of the characteristics of different types of network attacks in our model. For example, the feature ’dttl’ denotes destination to source time to live value, which provides insight into the network flow characteristics useful in detecting anomalous activities. Similarly, the UNSW NB15 feature ‘dwin’ refers to a destination TCP window size, which analyses the flow control behaviour of the destination host in TCP communications, enabling the detection of attacks such as DoS, buffer overflow and reconnaissance. The UNSW NB15 feature ‘dload’ refers to destination bits per second. By analysing the data transfer rate from the destination to the source, it helps in identifying unusual or malicious activities. By analysing the significance of these features, we may acquire insights into how the GCN-DQN classifier distinguishes between varieties of attacks and which features are important in these decisions. Finding the most informative features for intrusion detection can increase the accuracy of the classifier of our proposed GCN-DQN model. Moreover, it can offer insights into various attack types, facilitating more effective counter measures.

## 6. Conclusions

This paper presents a method for detecting malicious traffic, termed GCN-DQN, that integrates GCN and a self-attention mechanism with a Deep Q Network to enhance network anomaly detection. In our proposed model, we used GCN to make graph embeddings and attention mechanisms incorporate spatial and temporal features, as well as capturing intricate dependencies. The Deep Q Network integrated into our model significantly improves anomaly detection performance in our proposed GCN-DQN model. Experimental results using the publicly available UNSW-NB15 and CIC-IDS2017 datasets confirm that our approach enhances detection accuracy and lowers false alarm rates—an essential aspect of effective network traffic management. The proposed model achieved an accuracy of 99.02% on CIC-IDS2017, 97% on UNSW-NB15, and 92% for each dataset using the corresponding baseline models. The evaluation of our experimental test results showed that our model consistently performs better at identifying both normal and attack types, outperforming recent top models in accuracy, precision, recall, and F1 score.

Additionally, to enhance interpretability and build trust, our work employs global and local interpretations using SHAP and LIME, providing essential insights into the interpretability of the proposed IDS. Future research will concentrate on broadening the scope of NIDS to encompass other cross-domain applications, including fraud detection, SMEs anomaly identification, and personal security devices, which might substantially enhance its impact. Executing rigorous real-time deployment experiments to assess the actual performance of GCN-DQN-based NIDS across diverse network environments would more comprehensively enhance the understanding of its problems and advantages. We also aim to explore our proposed model on TinyML-based versions of GCN-DQN to enable execution on ultra-resource-constrained IoT devices.

## Figures and Tables

**Table 1:** The optimal hyperparameters of the GCN-DQN model.

UNSW-NB15		CIC-IDS2017	
Parameter	Value	Parameter	Value
num_epochs	250	num_epochs	250
learning rate	$1\times 10^{-3}$	learning rate	$1\times 10^{-3}$
batch_size	128	batch_size	128
dropout_rate	0.1	dropout_rate	0.3
num_heads	4	num_heads	4
gamma (DQN)	0.99	gamma (DQN)	0.99
epsilon (DQN)	1.0	epsilon (DQN)	1.0
epsilon_decay (DQN)	0.995	epsilon_decay (DQN)	0.995
epsilon_min (DQN)	0.01	epsilon_min (DQN)	0.01
weight_decay	$1\times 10^{-2}$	weight_decay	$1\times 10^{-2}$
label_smoothing	0.1	label_smoothing	0.1
early_stopping_patience	20	early_stopping_patience	20
gradient_clip_norm	1.0	gradient_clip_norm	1.0
optimizer	AdamW	optimizer	AdamW

**Table 2:** Binary classification results of the proposed model on the UNSW-NB15 dataset.

Class	Baseline Model				GCN-DQN			
	$A_{c}$	$P_{r}$	$R_{c}$	$F_{1}$	$A_{c}$	$P_{r}$	$R_{c}$	$F_{1}$
Normal	92.00	95.00	88.00	91.00	97.00	98.00	94.00	96.00
Attack	92.00	89.00	96.00	92.00	97.00	96.00	98.00	97.00

**Table 3:** Multiclass classification results of the proposed technique on CIC-IDS2017.

Class	Baseline Model				GCN-DQN			
	$A_{c}$	$P_{r}$	$R_{c}$	$F_{1}$	$A_{c}$	$P_{r}$	$R_{c}$	$F_{1}$
Normal	92.27	81.00	99.00	89.00	99.02	99.00	99.00	99.00
DoS hulk	92.27	99.00	79.00	88.00	99.02	99.00	99.00	99.00
Portscan	92.27	99.00	99.00	99.00	99.02	99.00	99.00	99.00

**Table 4:** Comparing our experimental results with other techniques on CIC-IDS2017.

Reference	Dataset	Approach	Acc%	XAI
[13]	UNSW NB15	SMOTE, Random Forest	95.1	×
[12]	UNSW NB15	IGRF-RFE, MLP	84.24	×
[36]	UNSW NB15	Stack Model [XGBoost KNN + XGBoost NN KNN]	96.2	×
[37]	CIC-IDS2017	CNN-BiLSTM-Attention	88.83	×
[30]	CIC-IDS2017	GNN, Continual learning (CL)	95.9	×
	UNSW NB15	GNN, Continual learning (CL)	96.4	×
[38]	UNSW NB15	GCN	96.40	×
	CIC-IDS2017	GCN	94.00	×
[39]	UNSW NB15	Wrapper-base feature selection, Multi-Head Attention-transformer	93.00	×
[40]	CIC-IDS2017	Transformer, Adversarial Autoencoder	97.3	×
Our Study	UNSW NB15	GCN-Multi-head attention, DQN	97.00	✓
	CIC-IDS2017	GCN-Multi-head attention, DQN	99.82	✓

## Data Availability

The datasets employed in this article are publicly available online at https://www.unb.ca/cic/datasets/ids-2017.html and https://research.unsw.edu.au/projects/unsw-nb15-dataset, accessed on 14 April 2025.
